# Comparative Genomics of *Exiguobacterium* Reveals What Makes a Cosmopolitan Bacterium

**DOI:** 10.1128/mSystems.00383-21

**Published:** 2021-07-20

**Authors:** Dechao Zhang, Zhaolu Zhu, Yangjie Li, Xudong Li, Ziyu Guan, Jinshui Zheng

**Affiliations:** a Institute of Oceanology, Chinese Academy of Sciences, Qingdao, China; b University of Chinese Academy of Sciences, Beijing, China; c Center for Ocean Mega-Science, Chinese Academy of Sciences, Qingdao, China; d State Key Laboratory of Agricultural Microbiology, Huazhong Agricultural University, Wuhan, China; e Hubei Key Laboratory of Agricultural Bioinformatics, College of Informatics, Huazhong Agricultural University, Wuhan, China; University of Illinois at Urbana-Champaign

**Keywords:** *Exiguobacterium*, cosmopolitan distribution, genomics, adaptation strategies, polysaccharide utilization, transporters

## Abstract

Although the strategies used by bacteria to adapt to specific environmental conditions are widely reported, fewer studies have addressed how microbes with a cosmopolitan distribution can survive in diverse ecosystems. *Exiguobacterium* is a versatile genus whose members are commonly found in various habitats. To better understand the mechanisms underlying the universality of *Exiguobacterium*, we collected 105 strains from diverse environments and performed large-scale metabolic and adaptive ability tests. We found that most *Exiguobacterium* members have the capacity to survive under wide ranges of temperature, salinity, and pH. According to phylogenetic and average nucleotide identity analyses, we identified 27 putative species and classified two genetic groups: groups I and II. Comparative genomic analysis revealed that the *Exiguobacterium* members utilize a variety of complex polysaccharides and proteins to support survival in diverse environments and also employ a number of chaperonins and transporters for this purpose. We observed that the group I species can be found in more diverse terrestrial environments and have a larger genome size than the group II species. Our analyses revealed that the expansion of transporter families drove genomic expansion in group I strains, and we identified 25 transporter families, many of which are involved in the transport of important substrates and resistance to environmental stresses and are enriched in group I strains. This study provides important insights into both the overall general genetic basis for the cosmopolitan distribution of a bacterial genus and the evolutionary and adaptive strategies of *Exiguobacterium*.

**IMPORTANCE** The wide distribution characteristics of *Exiguobacterium* make it a valuable model for studying the adaptive strategies of bacteria that can survive in multiple habitats. In this study, we reveal that members of the *Exiguobacterium* genus have a cosmopolitan distribution and share an extensive adaptability that enables them to survive in various environments. The capacities shared by *Exiguobacterium* members, such as their diverse means of polysaccharide utilization and environmental-stress resistance, provide an important basis for their cosmopolitan distribution. Furthermore, the selective expansion of transporter families has been a main driving force for genomic evolution in *Exiguobacterium*. Our findings improve our understanding of the adaptive and evolutionary mechanisms of cosmopolitan bacteria and the vital genomic traits that can facilitate niche adaptation.

## INTRODUCTION

Across the landscape, microbial communities are nonrandomly dispersed in terms of their composition and diversity (1). Physical and chemical factors in the environment significantly influence the distribution patterns of microbes (2). For example, marine and nonmarine habitats are separated by strong physiochemical differences in salinity, temperature, pH, dissolved oxygen, and water chemistry (3). As a result, most marine microbes belong to phylogenetic groups different from those of their freshwater and terrestrial relatives, and transitions between these two niches are rare (4, 5). It has frequently been reported that marine and freshwater/terrestrial bacterial members often utilize different strategies for niche adaptations (6). Comparative genomic analyses of ocean microbes have revealed that the genomes of many marine bacteria have been streamlined to reduce the metabolic costs of maintaining nonessential genetic material, which favors their adaptation to nutrient-poor ocean environments (7, 8). In contrast, free-living terrestrial bacteria usually have a normal genome size and exhibit frequent horizontal genetic-transfer events, which is believed to facilitate their capacity to use diverse nutrients and resist the stresses caused by complicated adverse environments (9, 10). The transition of a marine bacterium to a nonmarine bacterium or vice versa requires complex genomic evolution (11, 12). The gain and loss of genes involved in the transport, metabolism, and assimilation of different types of organic or inorganic nutrients are believed to play crucial roles during this process (11, 13). However, this knowledge is derived mainly from comparative genomic analyses of a few bacteria that are highly abundant in either marine or nonmarine microbiotas (14, 15). The strategies of evolution and adaptation for microbes that are widely distributed in both marine and nonmarine environments have not been well studied to date.

Bacteria of the genus *Exiguobacterium* are Gram-positive facultative anaerobes that have been frequently isolated from various habitats, including seawater, marine sediment, marine algae (16, 17), soil (18), freshwater (19), plant rhizosphere (20), and even extreme environments, such as a salt lake (21), glaciers, and hot springs (22). Previous studies also revealed that members of *Exiguobacterium* have transitioned between the terrestrial and marine niches (23, 24). Genomic analyses of these bacteria have provided some vital insights into their psychrophilic and thermophilic adaptations and resistances to multiple toxic compounds (25–28). However, we lack a comprehensive understanding of how these bacteria underwent evolutionary adaptation to marine and nonmarine habitats.

In this study, *Exiguobacterium* was used as a model to study the evolution and adaptive strategies of bacteria with a cosmopolitan distribution across diverse habitats. We first mined public databases for 16S rRNA gene sequences to reveal the diversity and distribution of members of this genus. We then isolated multiple strains from marine and terrestrial habitats and tested their adaptive and metabolic features. Furthermore, we sequenced the full genomes of 105 strains and performed large-scale phylogenomic and comparative genomic analyses using genomes of a total of 147 strains isolated from marine and nonmarine habitats worldwide. Special attention was paid to the genomic and metabolic characters that allow these organisms to respond to diverse environments.

## RESULTS

### *Exiguobacterium* members have a cosmopolitan distribution and share extensive abilities to adapt to survive in various environments.

**FIG 1:**
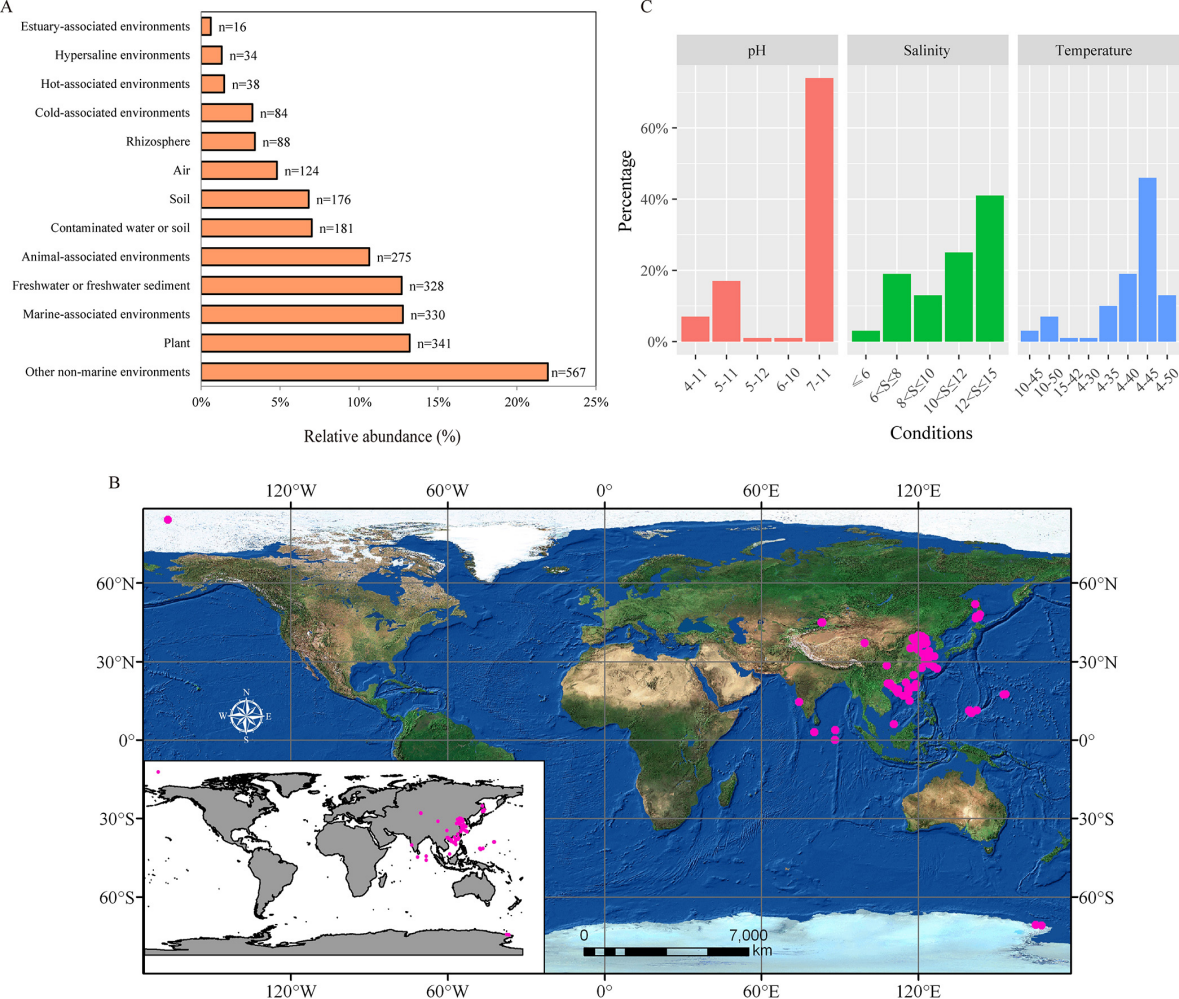
Cosmopolitan distribution of *Exiguobacterium* strains. (A) Relative abundances of various 16S rRNA gene sequences among 13 types of habitats. (B) Isolation sites (pink dots) of 97 *Exiguobacterium* strains examined in this study. The map was created with ArcGIS 10.6 software. (C) Temperature (°C), pH, and salinity (%) tolerance test results for 105 *Exiguobacterium* strains. The *x* axis represents the tolerance range of strains, and “S” represents salinity.

To explore the diversity and distribution of members belonging to the genus *Exiguobacterium*, we retrieved 16S rRNA gene sequences with >95% identities to those of reported type strains belonging to this genus from GenBank. A total of 2,582 *Exiguobacterium* 16S rRNA gene sequences with unambiguous information about isolation sources were collected (see Table S1 in the supplemental material). We found that members of this genus were frequently isolated from terrestrial environments (86.6%), including plants or rhizosphere (16.6%), animal skin or gut (10.7%), freshwater or freshwater sediments (12.7%), contaminated water or soil (7%), soil (6.8%), extreme environments (hot, cold, or hypersaline environments) (6%), air (4.8%), and other nonmarine environments (22%) (Fig. 1A; Table S1). The remaining members (13.4%) were isolated from marine-associated environments, including seawater, algae, and oceanic sediment. When we combined the location information, we found that *Exiguobacterium* can be found in ecosystems of all continents and oceans (Fig. S1A). This agrees with the current notion that *Exiguobacterium* is a cosmopolitan bacterial genus that includes many extremophiles capable of surviving in both marine and nonmarine environments worldwide (16).

10.1128/mSystems.00383-21.1 FIG S1 Geographic distribution of 16S rRNA gene sequences and pan-genome analysis. (A) Geographic distribution of 16S rRNA gene sequences with information about isolation sites (the pink dots represent the sampling locations, and the dots are colored by number of 16S rRNA gene sequences). The map was created using Tableau software (version 10.5) with map data from OpenStreetMap contributors. (B) Pan- and core genomes of the *Exiguobacterium* genus, i.e., the sizes of the pan- and core genomes in relation to numbers of genomes added into the gene pool. Box plots show the 25th and 75th percentiles, with medians shown as horizontal lines, and whiskers indicate the lowest and highest values within 1.5 times the interquartile range (IQR) from the first and third quartiles, respectively. The curve for the pan-genome is fitted by the power-law regression model, and the curve for the core genome is fitted by the exponential curve fit model. Download FIG S1, PDF file, 1.9 MB. Copyright © 2021 Zhang et al. 2021 Zhang et al. https://creativecommons.org/licenses/by/4.0/ This content is distributed under the terms of the Creative Commons Attribution 4.0 International license.

10.1128/mSystems.00383-21.2 TABLE S1 Information on the *Exiguobacterium* 16S rRNA gene sequences available in GenBank. Download Table S1, XLSX file, 0.10 MB. Copyright © 2021 Zhang et al. 2021 Zhang et al. https://creativecommons.org/licenses/by/4.0/ This content is distributed under the terms of the Creative Commons Attribution 4.0 International license.

To study the adaptation and evolution of *Exiguobacterium*, we performed large-scale phenotype tests and comparative genomics analysis. A total of 105 *Exiguobacterium* strains were collected here, including 86 isolates from marine niches (e.g., marine sediment, seawater, algae, marine cold springs, hydrothermal vents, seamounts, mangroves, marine fish, and coral), 11 strains from terrestrial environments (e.g., soil, salt lakes, coal mines, and pig farms), and eight type strains for which genomic data were previously unavailable (Fig. 1B; Table S2). We focused on strains isolated from marine habitats because most members of the *Exiguobacterium* genus with genomic data available in GenBank were from terrestrial environments.

10.1128/mSystems.00383-21.3 TABLE S2 Genome features and isolation sources of 147 *Exiguobacterium* strains. Download Table S2, XLSX file, 0.03 MB. Copyright © 2021 Zhang et al. 2021 Zhang et al. https://creativecommons.org/licenses/by/4.0/ This content is distributed under the terms of the Creative Commons Attribution 4.0 International license.

Previous studies showed that *Exiguobacterium* spp. can survive in a wide range of habitats, including cold, hot, hypersaline, and alkaline environments (17). However, it was unclear if such adaptability is shared by all members or if such capacities are strain/species specific. We thus performed large-scale phenotype tests and evaluated the growth potential of the 105 collected strains under different levels of pH, temperature, and salinity. We found that most *Exiguobacterium* members could survive and grow in a wide range of temperatures, salinities, and pH values (Fig. 1C; Table S3). The pH tests revealed that all strains were alkali resistant and able to survive in environments with pH values up to 11, and 24% of strains showed growth at pHs 5 and even 4. In the salinity test, most of the strains showed tolerance to salt, with 66% of strains showing growth in environments with NaCl concentrations above 10%. Our temperature tests showed that *Exiguobacterium* spp. can tolerate both high and low temperatures, with 91% of strains growing at 4°C and 61% surviving at high temperatures ranging from 40 to 50°C. We did not find any association between the growth abilities and source environments of analyzed strains. These results suggest that an extensive adaptability to survive in various environments is a general feature of this genus.

10.1128/mSystems.00383-21.4 TABLE S3 Temperature, pH, and salinity tolerance data for 105 *Exiguobacterium* strains. Download Table S3, XLSX file, 0.01 MB. Copyright © 2021 Zhang et al. 2021 Zhang et al. https://creativecommons.org/licenses/by/4.0/ This content is distributed under the terms of the Creative Commons Attribution 4.0 International license.

### Phylogenetic analysis identified two genetic groups in the *Exiguobacterium* genus.

**FIG 2:**
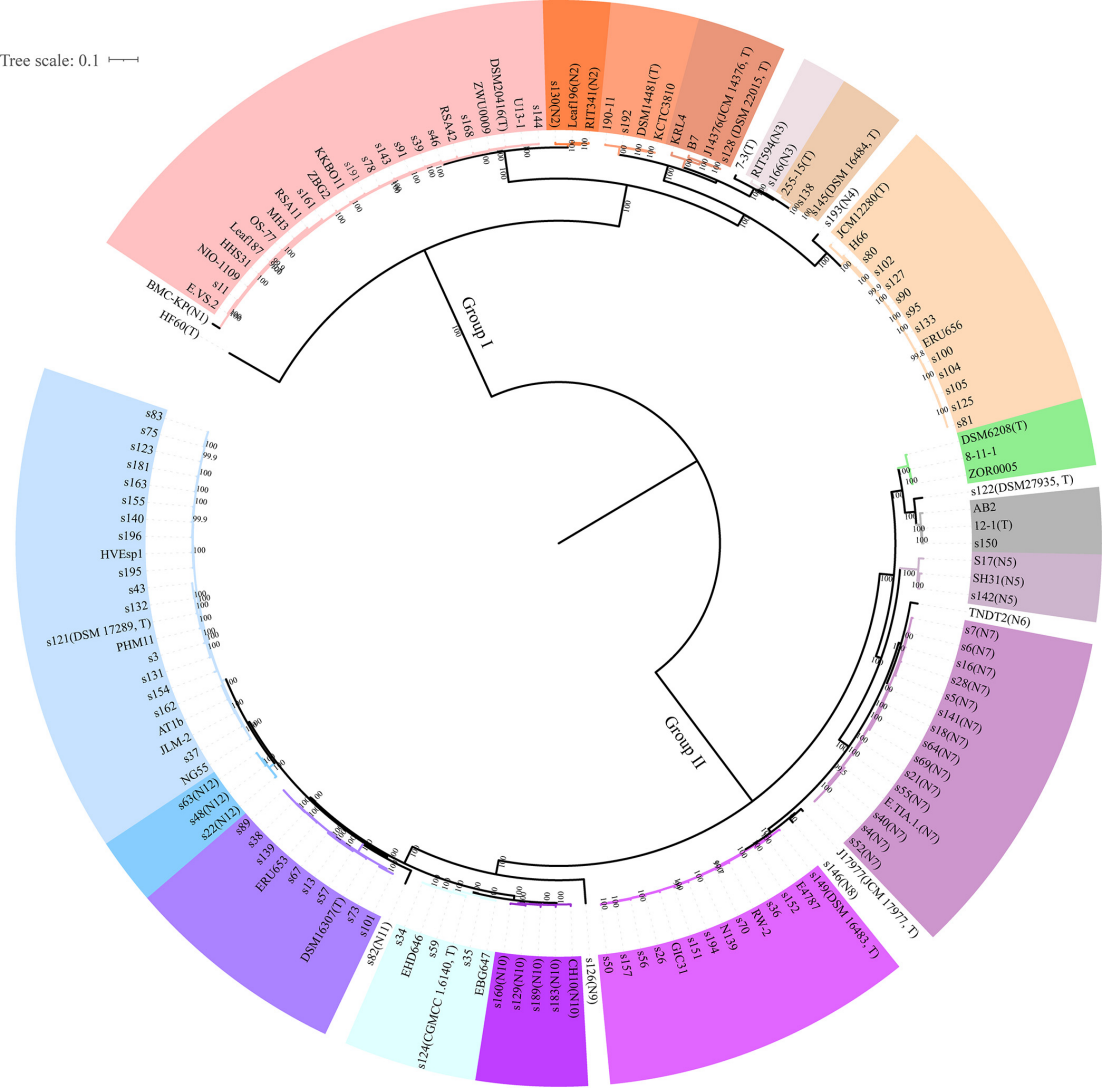
Phylogenetic analysis of *Exiguobacterium*. The tree was built using IQ-TREE based on the concatenated amino acid sequence alignments of single-copy core genes. Bootstrap support values were calculated from 1,000 replicates. “T” represents the type strain for the following: NIO-1109 for E. enclense, HHS31 for *E. indicum*, DSM20416 for *E. acetylicum*, DSM14481 for *E. undae*, J14376 for *E. soli*, s128 for *E. soli*, 7-3 for *E. sibiricum*, 255-15 for *E. sibiricum*, s145 for *E. artemiae*, JCM12280 for *E. oxidotolerans*, DSM6208 for *E. aurantiacum*, s122 for “*E. himgiriensis*,” 12-1 for *E. alkaliphilum*, J17977 for *E. aquaticum*, s149 for *E. mexicanum*, s124 for *E. aestuarii*, DSM16307 for *E. marinum*, and s121 for *E. profundum*. N1 to N12 represent putative new species. Different colors represent different putative species, which were differentiated using the threshold ANI of 95%.

We conducted comparative genomic analyses to investigate the strategies that these microbes have used in their evolution and adaptation to diverse environments. All genomes of the 105 collected *Exiguobacterium* strains were sequenced and assembled. Forty-two *Exiguobacterium* genomes available in GenBank with more than 95% genome completeness and less than 5% contamination were also included (Table S2). All predicted protein-coding genes from the 147 genomes were clustered into 8,857 groups; the 1,316 groups that were shared across all of the studied genomes were classified as core gene families. A maximum-likelihood (ML) phylogenetic tree was constructed based on the concatenated single-copy core gene alignment (Fig. 2). Two genetic groups of *Exiguobacterium* spp. were classified and were well supported by bootstrapping analysis; this grouping was consistent with that from a previous analysis based on 16S rRNA gene sequences of type strains (16). Using an average nucleotide identity (ANI) of 95% as the threshold to define different species (29), we classified the analyzed genomes into a total of 27 species, including 12 putative new species (N1 to N12) (Fig. 2; Table S4). Of them, 11 and 16 species belonged to groups I and II, respectively (Table S4).

10.1128/mSystems.00383-21.5 TABLE S4 Average nucleotide identity (ANI) analysis of 147 *Exiguobacterium* strains. Download Table S4, XLSX file, 0.1 MB. Copyright © 2021 Zhang et al. 2021 Zhang et al. https://creativecommons.org/licenses/by/4.0/ This content is distributed under the terms of the Creative Commons Attribution 4.0 International license.

We next annotated the tree by adding the isolation environment for each strain and found that strains from the same species could be found in different environments (Table S5). For example, strains of *E. acetylicum* were found in seawater, ocean sediment, soil, rhizosphere, a glacier, and even animal gut. This suggested that a given species of *Exiguobacterium* may colonize different niches of terrestrial and marine environments. This finding contrasts with the reported behavior of some typical marine bacteria, different lineages of which were found to adapt to marine versus nonmarine habits (30).

10.1128/mSystems.00383-21.6 TABLE S5 Niche distribution of 147 *Exiguobacterium* strains based on phylogenetic analysis. Download Table S5, XLSX file, 0.01 MB. Copyright © 2021 Zhang et al. 2021 Zhang et al. https://creativecommons.org/licenses/by/4.0/ This content is distributed under the terms of the Creative Commons Attribution 4.0 International license.

### Carbon and nitrogen source utilization for wide adaptation.

**FIG 3:**
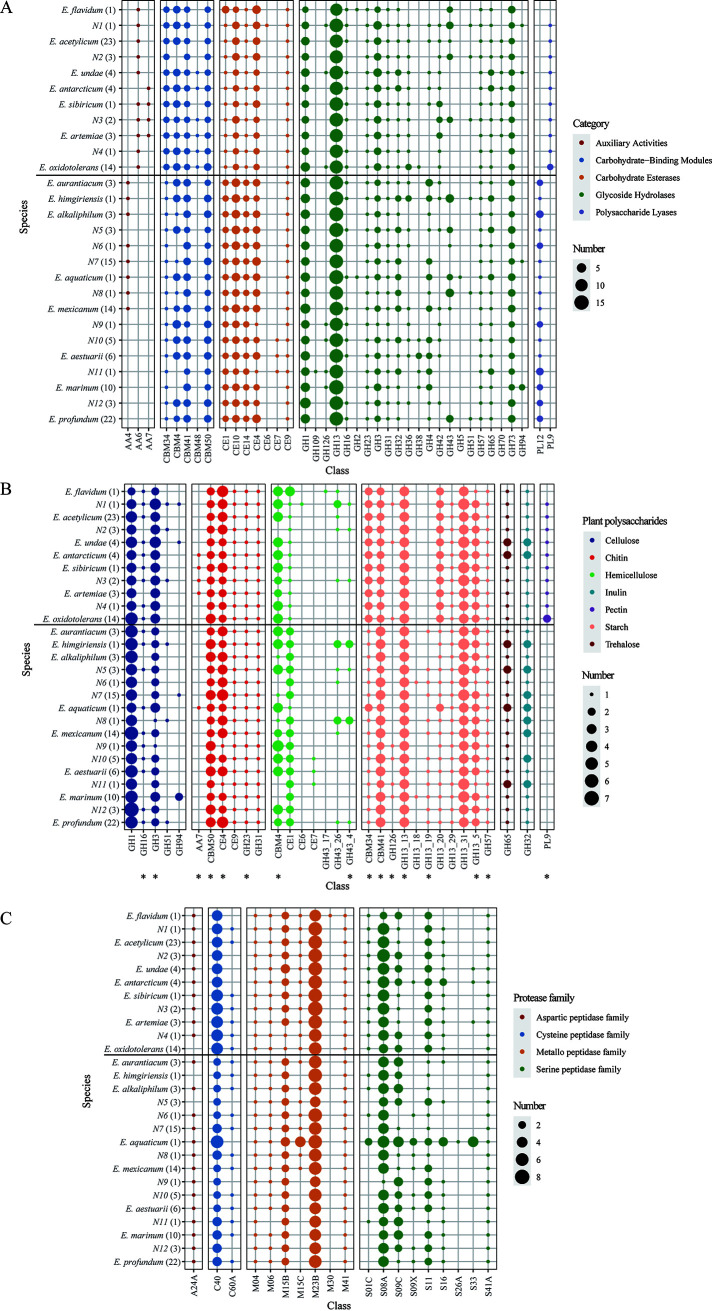
Carbon and nitrogen source utilization. (A to C) Numbers of carbohydrate-active enzymes (CAZymes) (A), plant polysaccharide degradation enzymes (B), and extracellular peptidases (C) encoded in *Exiguobacterium* genomes. Parentheses enclose the total number of genomes in each *Exiguobacterium* species. Asterisks indicate CAZymes with a potential secretion signal. The thick black thick line separates the species list into two parts; the species above the line belong to group I, while those below the line belong to group II.

To explore a genetic basis for the ability of *Exiguobacterium* strains to survive in different niche types, we first focused on genes involved in nutrient metabolism. Carbohydrate-active enzymes (CAZymes) are involved in carbohydrate metabolism. A total of 7,976 genes belonging to five CAZyme superfamilies were identified from all of the studied genomes, with 61.5%, 18.7%, 15.9%, 2.7%, and 1.2% of these genes corresponding to glycoside hydrolase (GH), carbohydrate esterase (CE), the carbohydrate-binding module (CBM), polysaccharide lyase (PL), and auxiliary activities (AA), respectively (Fig. 3A; Table S6). Each of the studied genomic sequences encoded 43 to 68 of these enzymes.

10.1128/mSystems.00383-21.7 TABLE S6 Gene number of carbohydrate-active enzymes (CAZymes) and extracellular peptidases detected in each *Exiguobacterium* genome. Download Table S6, XLSX file, 0.1 MB. Copyright © 2021 Zhang et al. 2021 Zhang et al. https://creativecommons.org/licenses/by/4.0/ This content is distributed under the terms of the Creative Commons Attribution 4.0 International license.

Seven classes of enzyme for complex polysaccharide degradation were predicted from *Exiguobacterium* genomes (Fig. 3B; Table S6). The top three most numerous classes were those associated with the degradation of starch, cellulose, and chitin, and most of these CAZymes were found to have the potential to be secreted (Fig. 3B). Family GH13 represented the main amylolytic enzyme family; among the members of this family, GH13_31 (α-glucosidase) and GH13_13 (pullulanase) were the top two most numerous subfamilies (3.3 and 3 genes per genome, respectively). The most numerous family involved in cellulose degradation was GH1 (β-glucosidases, with more than 4 genes per genome). For chitin degradation, family CE4 (deacetylase) and CBM50 (chitin binding) showed significantly higher abundances.

Proteinaceous compounds are abundant forms of organic nitrogen that are found in aquatic and soil environments (31). Extracellular microbial peptidases are very important in both marine and terrestrial environments, as they play a crucial role in degrading organic nitrogen and thereby contribute to global nitrogen cycling (31). In this work, 3,912 putatively secreted peptidases were identified and assigned to 20 families; of them, 43.7%, 38.1%, 15%, and 3.2% belonged to the metallo, serine, cysteine, and aspartic peptidase families, respectively (Fig. 3C; Table S6). When normalized to the genome size, the average number of secreted peptidase-coding genes was nine genes per Mb, which is higher than the number (5.84) found in bacteria overall (31). Among these secreted peptidases, the metallo peptidase M23 and serine peptidase S08 represented the top two most abundant peptidases (Fig. 3C).

To validate the potential ability of *Exiguobacterium* spp. to degrade and metabolize complex carbohydrates and proteins, we tested the amylase and protease activities of the 105 studied strains on plates (Table S7). All of the strains effectively hydrolyzed starch, and approximately 70% could degrade proteins. Together, the results from our genomic analysis and activity testing provide strong evidence that most members of the *Exiguobacterium* genus can metabolize and utilize a wide range of polysaccharides and proteins from marine and nonmarine environments. This likely explains the genetic basis for the cosmopolitan distribution of these bacteria.

10.1128/mSystems.00383-21.8 TABLE S7 Validation of the abilities of *Exiguobacterium* spp. to degrade and metabolize complex carbohydrates and peptides. Download Table S7, XLSX file, 0.01 MB. Copyright © 2021 Zhang et al. 2021 Zhang et al. https://creativecommons.org/licenses/by/4.0/ This content is distributed under the terms of the Creative Commons Attribution 4.0 International license.

### Genetic basis of maintaining homeostasis in extreme environments.

**FIG 4:**
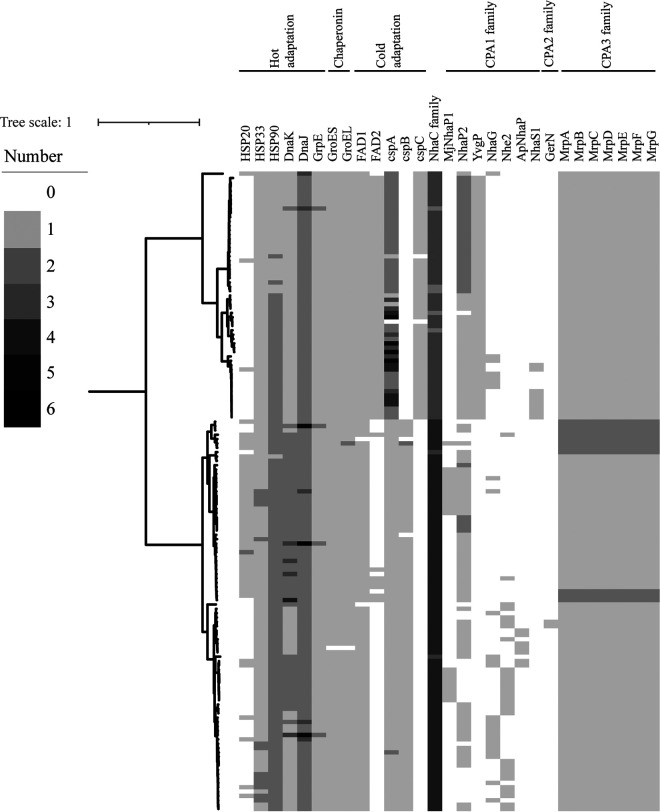
Genes detected across *Exiguobacterium* genomes are vital for maintaining homeostasis in extreme environments. The heatmap represents the vital gene numbers and their distribution across the 147 genomes. The maximum-likelihood tree was constructed by IQ-TREE as described in Materials and Methods.

Bacteria use two main strategies to survive in cold environments; they use branched-chain fatty acids to maintain membrane fluidity and express cold shock proteins (Csps) that stabilize the bacterial cytosol at low temperatures (32, 33). Our genomic analysis suggested that all members of the *Exiguobacterium* genus could use both strategies to cope with low temperature. Two types of fatty acid desaturase (FAD) involved in unsaturated branched-chain fatty acid production were identified in *Exiguobacterium* genomes (Fig. 4; Table S8). All genomes except those of AB2 and s126 encoded at least one FAD1 protein, while genes for FAD2 were found mainly in strains belonging to group I. FADs can synthesize unsaturated fatty acids to maintain membrane fluidity at low temperatures, and potential cold-induced changes in the metabolic pathway of fatty acids were previously identified in E. antarcticum B7 (32–34). Three types of *csp* (*cspA*, *cspB*, and *cspC*) were predicted from all of the studied genomes (Fig. 4). Most genomes of group I members contained more than two *cspA* genes, while those from group II had only one, and *cspB* and *cspC* were specifically found in members of groups I and II, respectively. Csps are important for stabilizing the bacterial cytosol in cold environments and are induced upon a temperature downshift (35). It is worth noting that the psychrotrophic (able to grow in cold environments) strains were clustered mainly in group I; *fad2* and *cspA*, enriched in group I, may contribute to the difference.

10.1128/mSystems.00383-21.9 TABLE S8 Genetic basis for adaptation of *Exiguobacterium* strains to diverse habitats. Download Table S8, XLSX file, 0.03 MB. Copyright © 2021 Zhang et al. 2021 Zhang et al. https://creativecommons.org/licenses/by/4.0/ This content is distributed under the terms of the Creative Commons Attribution 4.0 International license.

To survive in hot environments, *Exiguobacterium* spp. contain the heat shock gene cluster *grpE-dnaJ*-*dnaK* (Fig. 4; Table S8). DnaK plays a major role in maintaining protein homeostasis under thermal-stress conditions, and DnaJ and GrpE are cochaperones for DnaK (36). GroEL/GroES can reportedly cooperate with DnaK/DnaJ to prevent protein misfolding in bacteria (37). Thus, it is relevant that genes encoding GroEL and GroES were also discovered in all of the studied strains, with the exception of EHD646 (Fig. 4; Table S8). Both DnaK/DnaJ/GrpE and GroEL/GroES were reported to prevent aggregation and denaturation of proteins at high temperature in bacteria (38). Three types of heat shock proteins (HSPs; HSP20, HSP33, and HSP90) were also predicted. These HSPs can prevent irreversible protein denaturation and are important chaperones that help mediate appropriate responses to heat or oxidative stress (39–42). Together, the previous and present findings suggest that members of this genus utilize multiple strategies to cope with hot environments.

In bacteria, the Na^+^:H^+^ antiporters play crucial roles in maintaining intracellular pH homeostasis and the dynamic balance of cellular Na^+^. According to the Transporter Classification Database (TCDB), Na^+^:H^+^ antiporters contain mainly members of the large monovalent cation/proton antiporter (CPA) family, such as CPA1, CPA2, and CPA3, and the NhaC Na^+^:H^+^ antiporter family (43, 44). Seven types of CPA1 and one CPA2 were predicted from the genomes of *Exiguobacterium* spp. (Fig. 4; Table S8); the former were more frequently found in group I members, and the latter were more common in group II members. Compared to CPA1 and CPA2 antiporters, CPA3 antiporters are more structurally complex, as they have a multicomponent structure consisting of either seven or six members (45). The multicomponent Na^+^:H^+^ antiporter (Mrp) of CPA3 has been shown to provide Na^+^/H^+^ antiporter activity and function in the multiple compound resistance and pH homeostasis processes of Bacillus subtilis (45). In the present study, Mrp antiporters were identified in all *Exiguobacterium* genomes (Fig. 4; Table S8). In addition, an antiporter from the NhaC Na^+^:H^+^ antiporter (NhaC) family was identified in all *Exiguobacterium* genomes with copy numbers up to six (Fig. 4; Table S8). The presence of multiple types of Na^+^:H^+^ antiporter likely provides the basis for *Exiguobacterium* strains to maintain their osmotic and pH balances under various environments.

Together, the existence of diverse important proteins, including cold shock proteins, heat shock proteins, chaperonins, fatty acid desaturase, and diverse Na^+^:H^+^ antiporters, may explain the broad range of temperatures, pH, and salinity in which *Exiguobacterium* strains can survive to colonize diverse habitats.

### Transporter expansion has driven the genomic expansion of *Exiguobacterium* spp.

We found that strains from marine environments were more frequently assigned to group II, while species from group I had more diverse terrestrial-niche distributions. Our analysis of 16S rRNA gene sequences from GenBank showed that of the 346 members from marine environments, 54 and 292 belonged to groups I and II, respectively (Table S1). Of the 86 marine strains isolated in this study, 24 and 62 belonged to groups I and II, respectively. Of the 11 species classified to group I, 10 contained strains that were isolated from both marine and terrestrial (e.g., soil, plant rhizosphere, fresh water, etc.) environments (Table S5). In group II, in contrast, most of the species were isolated solely from marine environments (Table S5).

**FIG 5:**
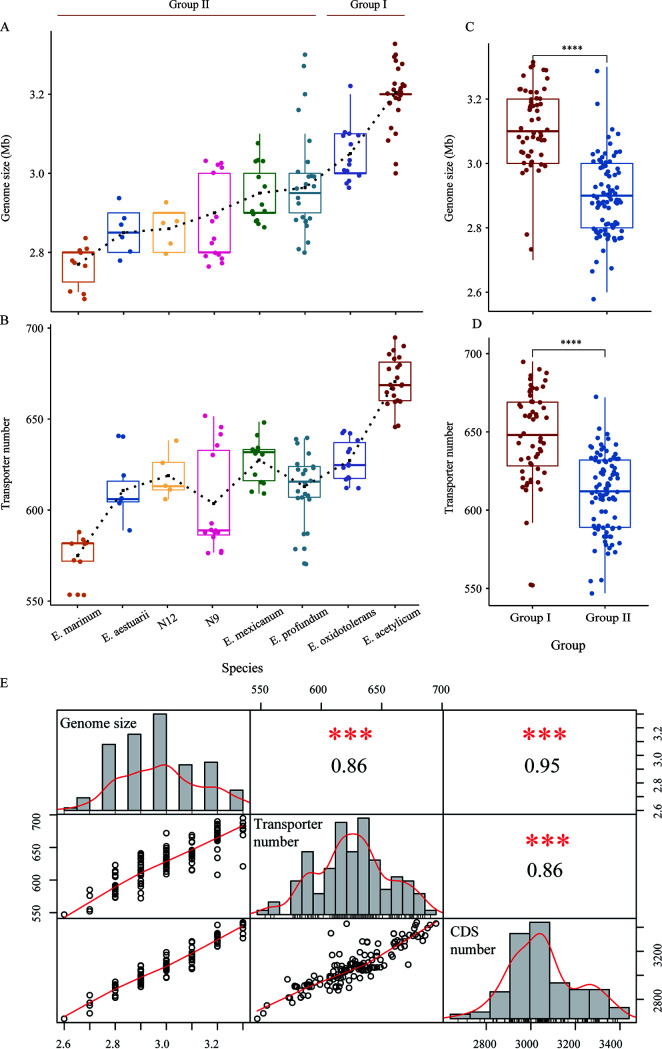
Comparison and Spearman’s correlation analysis of genome sizes and transporter numbers. (A) Trends in the genome sizes of *Exiguobacterium* species (species with at least 5 strains were selected to show the trends). (B) Trends in the transporter numbers of *Exiguobacterium* species (species with at least 5 strains were selected to show the trends). (C) Comparison of genome sizes between group I and group II. (Black ****, significantly different at a *P *of <0.0001, as assessed by Wilcoxon tests). (D) Comparison of transporter numbers between group I and group II. (E) Spearman’s correlation analysis of genome sizes, CDS numbers, and transporter numbers. The genome size, CDS number, and transporter number are shown in the central diagonal, and the scatterplots are depicted with a fitted red line. On the corresponding side for each pairing is the Spearman rank correlation coefficient, *r_s_*. (Red ***, *P < *0.01).

To understand the genetic background underlying the ecological differences of the two groups, we performed comparative genomic analysis. We found that the genome size and transporter number tended to increase for species of group I relative to those for group II (Fig. 5A and B and Table S9). The average genome size and transporter number of group I (3.12 Mb and 648, respectively) were significantly larger than those of group II (2.90 Mb and 610, respectively) (*P < *0.0001 by the Wilcoxon test) (Fig. 5C and D). A pan-genome analysis suggested that there is a high level of genomic plasticity in this genus (Fig. S1B). The presence of an open pan-genome in *Exiguobacterium* spp. suggests that these species can change their genomes to facilitate their adaption to different habitats.

10.1128/mSystems.00383-21.10 TABLE S9 Transporter analysis of 147 *Exiguobacterium* genomes. Download Table S9, XLSX file, 0.2 MB. Copyright © 2021 Zhang et al. 2021 Zhang et al. https://creativecommons.org/licenses/by/4.0/ This content is distributed under the terms of the Creative Commons Attribution 4.0 International license.

**FIG 6:**
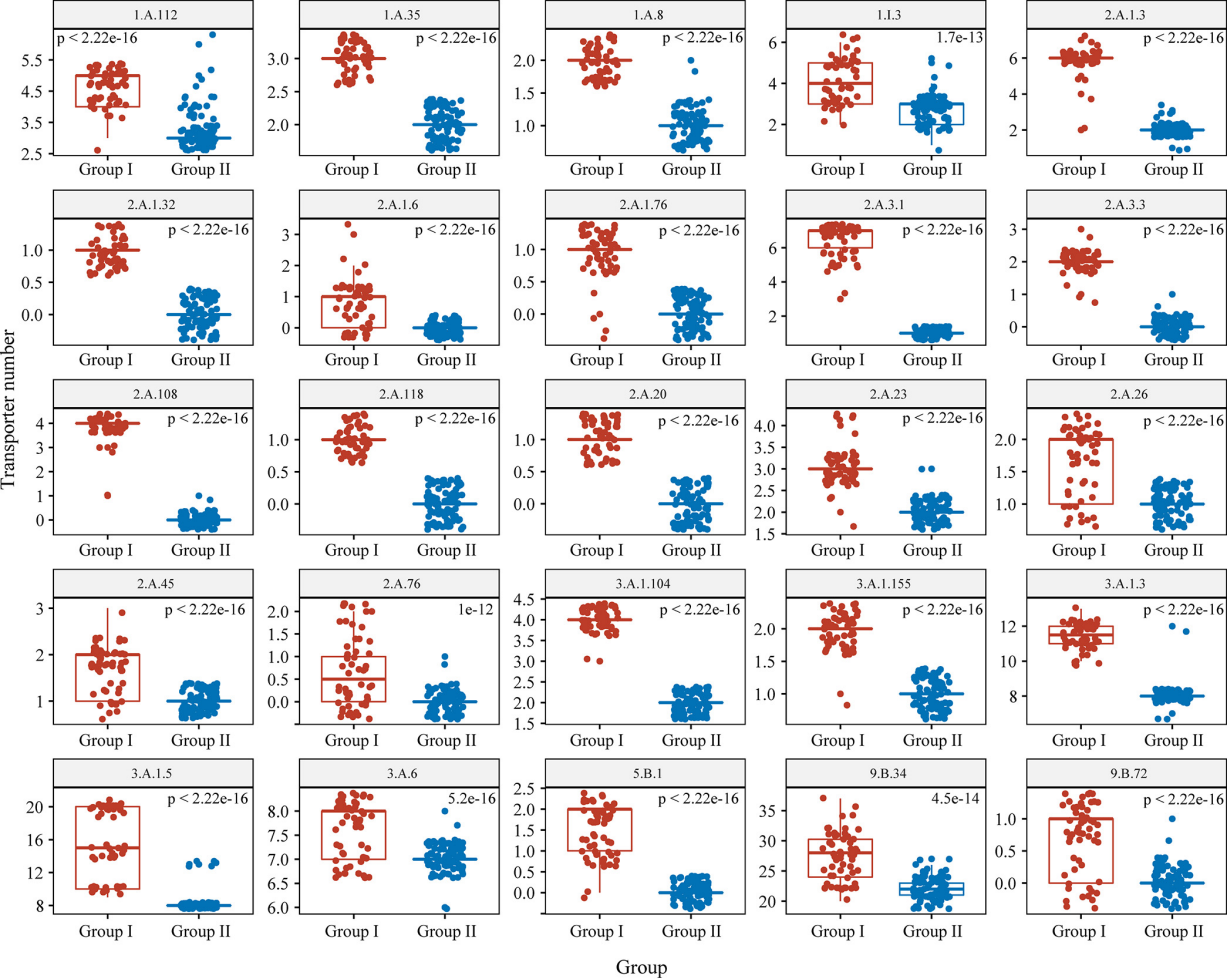
Comparison of 25 transporter family members between groups I and II. All pairwise comparisons were significantly different (Wilcoxon test). Each dot represents one strain.

Spearman’s coefficient obtained for the correlation of transporter number with genome size was 0.86 (Fig. 5E). A significant correlation (*P < *0.01) was found between genome size and the total number of transporter genes, suggesting that the expansion of transporters may have contributed to the difference in genomic contents between group I and group II strains. Among the 247 identified transporter families, 25 were predicted to have high degrees of correlation between their numbers for both genome size and protein coding sequence (CDS) (Spearman’s correlation coefficient > 0.6, *P < *0.01) (Table 1). These 25 transporter gene families were significantly enriched in the genomes of group I members compared to group II members (Fig. 6; Table S9). Among these families, seven are associated with the transport of diverse amino acids, such as cationic, polar, branched-chain, and basic amino acids (Table 1), which are important components for nitrogen metabolism, protein synthesis, cell growth, and energy production or conversion (46). In addition, 2 of the 25 families are involved in the transport of Mg^2+^, namely, the cyclin M Mg^2+^ exporter family and the CorA metal ion transporter family. Mg^2+^ homeostasis is important in bacteria and has been reported to play a critical role in their thermotolerance (47, 48). The inorganic phosphate transporter family was also found to be expanded in group I relative to group II (Table 1). Phosphorus (P) is commonly associated with oxygen in the form of inorganic phosphates (PO_4_^−^ and P_i_) in the environment, and it is a crucial element involved in many energy production and metabolic pathways in bacteria (49).

**TABLE 1:** Correlation analysis of transporter family with both genome size and CDS number^*a*^

Genome content	TC no.	Family	Correlation coefficient	*P* value
Genome size	2.A.1	**Major facilitator superfamily (MFS)**	0.73	0
CDS no.	2.A.1		0.66	0
Genome size	2.A.1.3	Drug:H^+^ antiporter-2 (14-spanner) (DHA2) family	0.73	0
CDS no.	2.A.1.3		0.66	0
Genome size	2.A.1.76	Uncharacterized major facilitator 24 family	0.69	0
CDS no.	2.A.1.76		0.61	4.44E−16
Genome size	2.A.1.32	Putative aromatic compound/drug exporter (ACDE) family	0.68	0
CDS no.	2.A.1.32		0.60	8.88E−16
Genome size	2.A.1.6	Metabolite:H^+^ symporter (MHS) family	0.66	0
CDS no.	2.A.1.6		0.67	0
Genome size	**2.A.3**	**Amino acid-polyamine-organocation (APC) superfamily**	0.67	0
CDS no.	**2.A.3**		0.61	2.22E−16
Genome size	2.A.3.1	Amino acid transporter (AAT) family	0.71	0
CDS no.	2.A.3.1		0.66	0
Genome size	2.A.3.3	Cationic amino acid transporter (CAT) family	0.68	0
CDS no.	2.A.3.3		0.60	8.88E−16
Genome size	**3.A.1**	**ATP-binding cassette (ABC) superfamily**	0.75	0
CDS no.	**3.A.1**		0.69	0
Genome size	3.A.1.5	Peptide/opine/nickel uptake transporter (PepT) family	0.72	0
CDS no.	3.A.1.5		0.68	0
Genome size	3.A.1.3	Polar amino acid uptake transporter (PAAT) family	0.73	0
CDS no.	3.A.1.3		0.66	0
Genome size	3.A.1.155	Phage infection protein (PIP) family	0.72	0
CDS no.	3.A.1.155		0.63	0
Genome size	3.A.1.104	Teichoic acid exporter (TAE) family	0.68	0
CDS no.	3.A.1.104		0.60	1.33E−15
Genome size	1.A.112	Cyclin M Mg^2+^ exporter (CNNM) family	0.72	0
CDS no.	1.A.112		0.70	0
Genome size	1.A.8	Major intrinsic protein (MIP) family	0.70	0
CDS no.	1.A.8		0.61	2.22E−16
Genome size	1.A.35	CorA metal ion transporter (MIT) family	0.68	0
CDS no.	1.A.35		0.60	8.88E−16
Genome size	1.I.3	Bacterial (*Planctomycetes*) nuclear pore-like complex (B-NPC) family	0.63	0
CDS no.	1.I.3		0.62	2.22E−16
Genome size	2.A.23	Dicarboxylate/amino acid:cation (Na^+^ or H^+^) symporter (DAACS) family	0.71	0
CDS no.	2.A.23		0.65	0
Genome size	2.A.26	Branched-chain amino acid:cation symporter (LIVCS) family	0.61	4.44E−16
CDS no.	2.A.26		0.61	2.22E−16
Genome size	2.A.45	Arsenite-antimonite (ArsB) efflux family	0.60	1.11E−15
CDS no.	2.A.45		0.62	0
Genome size	2.A.118	Basic amino acid antiporter (ArcD) family	0.68	0
CDS no.	2.A.118		0.60	8.88E−16
Genome size	2.A.20	Inorganic phosphate transporter (PiT) family	0.68	0
CDS no.	2.A.20		0.60	8.88E−16
Genome size	2.A.108	Iron/lead transporter (ILT) family	0.69	0
CDS no.	2.A.108		0.61	4.44E−16
Genome size	2.A.76	Resistance to homoserine/threonine (RhtB) family	0.61	8.88E−16
CDS no.	2.A.76		0.61	4.44E−16
Genome size	3.A.6	Type III (virulence-related) secretory pathway (IIISP) family	0.62	0
CDS no.	3.A.6		0.62	0
Genome size	5.B.1	gp91^phox^ phagocyte NADPH oxidase-associated cytochrome *b*_558_ (Phox) family	0.73	0
CDS no.	5.B.1		0.67	0
Genome size	9.B.34	Kinase/phosphatase/cyclic GMP synthase/cyclic di-GMP hydrolase (KPSH) family	0.75	0
CDS no.	9.B.34		0.76	0
Genome size	9.B.72	4 TMS GlpM (GlpM) family	0.63	0
CDS no.	9.B.72		0.63	0

a TC, transporter classification. Boldface indicates superfamily.

The 25 selected transporter families also included three families involved in transporting heavy metal ions (the arsenite-antimonite efflux family, iron/lead transporter family, and peptide/opine/nickel uptake transporter family) and two major facilitator superfamilies (MFSs) associated with drug efflux (Table 1). These transporters have been shown to counteract the effects of toxic heavy metals and drugs (28, 50–52). Two of the 25 families are involved in the formation of the bacterial cell wall and biofilm, namely, the teichoic acid exporter family and the 4 TMS GlpM family. Teichoic acid is a major cell wall component of Gram-positive bacteria that plays crucial roles in bacterial resistance to antimicrobial agents and survival under disadvantageous conditions (53, 54). The 4 TMS GlpM family is required for normal production of alginate (55). Alginates are important polymeric substances that contribute to forming and developing the biofilm matrixes of numerous bacteria and enhancing colonization and thereby persistence under environmental stresses (56).

In all living organisms, transporters are vital to the uptake of nutrients, secretion of metabolites, maintenance of ion concentration gradients across membranes, and efflux of drugs and toxins (57). The enhanced ability of group I strains for transporting important substrates enables such strains to better meet demands for cellular metabolism and functions and thus appears to provide an important basis for their ability to survive in diverse terrestrial environments. Our results further suggest that the selective expansion of transporter families has been a major evolutionary strategy for members of the genus *Exiguobacterium*.

## DISCUSSION

The *Exiguobacterium* genus comprises Gram-positive, non-spore-forming, and facultative anaerobic species. Members belonging to this genus have been isolated from a large array of environments, such as soils (18), freshwater (19), plant rhizospheres (20), salt lake sediment (21), a hot spring (22), glaciers, and permafrost (58). 16S rRNA marker gene surveys have uncovered *Exiguobacterium* spp. surviving in even more diverse habitats, including indoor air (59), skin (60), fish gut (61), and a cold desert (62). These results suggest that members of the genus *Exiguobacterium* are ubiquitous. In this study, we first explored the distribution of *Exiguobacterium* members through 16S rRNA gene analysis and correlated that with information on the collection location. We found that members of the *Exiguobacterium* genus are distributed in both marine and terrestrial environments worldwide but that GenBank contains more from terrestrial environments than from marine environments. We next set out to isolate additional *Exiguobacterium* members from marine ecosystems. We obtained 86 isolates from diverse marine niches, including marine sediment, seawater, algae, cold springs, hydrothermal vents, seamounts, mangroves, marine fish, and coral. This suggests that *Exiguobacterium* spp. are widespread in a variety of marine environments and, together with the existing data, shows that members of the *Exiguobacterium* genus have a cosmopolitan distribution.

Our comprehensive experimental results indicate that members of this genus have a wide range of metabolic and stress resistance capabilities, which may contribute to their wide distribution. The ability to degrade and absorb carbon and nitrogen nutrients is crucial for the ability of a microorganism to survive in diverse habitats (63). Genes encoding multiple types of proteins capable of directing carbohydrate hydrolysis were identified in each *Exiguobacterium* genome, suggesting that these bacteria have versatile abilities to metabolize carbohydrates. Among the predicted enzymes, those involved in degrading starch, cellulose, and chitin were enriched in almost all studied members of the *Exiguobacterium* genus. Starch is an important storage polysaccharide for plants of both terrestrial and marine environments (64, 65), Cellulose, which is the most prevalent polysaccharide in nature, makes up the cell walls of plants and algae (66, 67). Chitin, which is the second-most-common polysaccharide in nature (after cellulose), is also widely distributed in terrestrial and marine ecosystems; it functions as a major structural component of crustacean shells, arthropod exoskeletons, and diatom cell walls (68–70). The presence of abundant genes for chitin degradation may enable members of the *Exiguobacterium* genus to occupy different functional niches with regard to chitin breakdown. The potential to utilize the most plentiful polysaccharides from both marine and terrestrial environments likely supports the ability of *Exiguobacterium* members to survive and reproduce under an extensive range of environmental conditions. It is also notable that many secreted peptidases are predicted in *Exiguobacterium* genomes. As proteinaceous compounds are important nitrogen nutrients for microorganisms (71), the presence of numerous potentially secreted peptidases in *Exiguobacterium* genomes may allow them to exploit different niches for nitrogen source uptake in different environments. The metallo peptidase M23 and serine peptidase S08 were the most abundant peptidases. M23 peptidase reportedly degrades bacterial extracellular peptidoglycans and thereby contributes to the acquisition of nutrition or defense against competitors (72, 73). Serine peptidases are often used as marker enzymes for proteolytic activity in soil and play important roles in the ability of a microorganism to utilize nitrogen sources in the environment (74). The capacity of *Exiguobacterium* spp. to metabolize starches and proteins was validated on plates, further supporting our proposal that such genes form at least part of the genetic basis for the cosmopolitan distribution of these bacteria.

In addition to being collected from the common terrestrial and marine ecosystems, many *Exiguobacterium* isolates have been collected from extreme environments, such as glaciers, hot springs, and salt lakes (17). Thus, members of the *Exiguobacterium* genus can be classified as extremophiles. Consistently with this, our analysis showed that the genomes of *Exiguobacterium* spp. contain genes related to various classes of stress resistance systems and that members often have more than one strategy to fight against certain extreme environments. Here, we predict that *Exiguobacterium* spp. may use two strategies to survive in cold environments: the maintenance of membrane fluidity and the stabilization of their cytosol. To cope with hot environments, they appear to use two types of chaperone systems to prevent aggregation and denaturation of proteins at high temperature. Moreover, three types of antiporter families are utilized to maintain intracellular pH homeostasis and the dynamic balance of cellular Na^+^. In general, a bacterium is adapted to cope with only one particular extreme environment. For instance, members of the genus *Psychrobacter* have vital CSPs and thus are more frequently found in cold environments (75). The presence of multiple stress-responsive genes discussed herein might enable strains of *Exiguobacterium* spp. to adapt to a very wide range of niches, even extreme environments.

Although the members of the genus *Exiguobacterium* have a cosmopolitan distribution overall, we herein uncovered ecological differences related to the two groups defined for this genus based on our genomic analysis. More numerous transporters and a more diverse nonmarine environmental distribution were found for group I, which may suggest that the expansion of transporters contributes to these ecological differences. Most of the expanded transporter families were associated with important physiological metabolism and environmental-stress resistance processes. Compared with the marine environment, the terrestrial environment is more diverse and has a greater variety of complex microenvironments due to the influence of climates or seasons, leading to greater diversity in the experienced stress conditions and nutrient substrates (3, 76). In order to survive in these more diverse environments, bacteria must develop specific systems for survival, such as nutrient-sensing and transport systems (77). Efficient transport of substances related to metabolism, cellular function, and/or environmental-stress resistance is crucial for bacterial survival in a variety of environments (78). Therefore, the expanded families that contribute to environmental-stress resistance and the transport of organic or inorganic substrates may play crucial roles in the ability of *Exiguobacterium* species group I members to survive in more diverse terrestrial environments.

### Conclusions.

The question of how microbes with a cosmopolitan distribution adapt to diverse habitats is an important topic in microbial ecology. Their wide distribution makes the genus *Exiguobacterium* a valuable system for studying the evolution and adaptive strategies that bacteria use to survive in multiple habitats. We herein reveal that *Exiguobacterium* spp. share extensive characteristics that enable them to adapt to various environments and exhibit a cosmopolitan distribution. Our genomic analysis revealed that *Exiguobacterium* members not only can utilize a variety of complex polysaccharides and proteins that are ubiquitous in both terrestrial and marine environments but also have a number of chaperonins and transporters that are expected to help them to survive in diverse extreme environments. We also revealed that the expansion of transporter families has driven genomic evolution in *Exiguobacterium*. The selective expansion of genes involved in transporting organic or inorganic substrates and environmental-stress resistance appears to have served as an evolutionary and adaptive strategy that has supported the extensive distribution of *Exiguobacterium* spp.

## MATERIALS AND METHODS

### Analysis of 16S rRNA gene sequences of *Exiguobacterium* spp.

The 16S rRNA gene sequences of *Exiguobacterium* spp. available in GenBank were retrieved. Sequences that had low quality (e.g., sequences that were not ribosomal or that contained N in the base sequence) or lacked isolation source tags were discarded, and 16S rRNA gene sequences with >95% identities to those of reported type strains were retained. Information on the isolation source of these sequences was collected, and the habitats were classified into 13 types: air, animal-associated environments, cold environments, estuary-associated environments, contaminated water or soil, freshwater or freshwater sediment, hot environments, hypersaline environments, marine environments, plants, rhizospheres, soil, and other inland environments.

### Bacterial isolation, culture, and identification.

In this study, we collected a total of 105 strains, including 97 isolates that our group collected from terrestrial and marine environments worldwide and eight type strains obtained from the DSMZ, the Japan Collection of Microorganisms (JCM), and the China General Microbiological Culture Collection Center (CGMCC) (see Table S2 in the supplemental material). To obtain each target strain, initial samples from marine and terrestrial environments were macerated and mixed with sterile saline solution (0.8%) using a standard dilution plating method on marine agar 2216 (MA; Difco) and LB agar at 20°C. Each plate-grown colony was picked and subcultured three times to achieve a pure culture. 16S rRNA sequence analysis was performed to identify those belonging to the *Exiguobacterium* genus. All of the 105 strains were confirmed to grow in sea salt-free medium and were routinely cultivated on LB agar and in liquid LB for subsequent genomic sequencing.

### Adaptive ability tests for pH, temperature, and salinity.

To assess the range of adaptation to pH, temperature, and salinity, all 105 strains were monitored for growth under the following conditions: for temperature-based assessments, at 4, 10, 25, 30, 40, 45, and 55°C on LB agar and in liquid LB at 150 rpm; for pH-based assessments, at 25°C in liquid LB medium in 1-unit steps from pH 4.0 to pH 11.0 using citrate-Na_2_HPO_4_ buffer (pH 4.0 to 7.0), Tris buffer (pH 7.5 to 9.0), and NaHCO_3_-Na_2_CO_3_ buffer (pH 9.5 to 11.0); and for salinity assessments, at 0 to 20% (in increments of 1%, wt/vol) NaCl after 2 to 3 days of cultivation at 25°C in medium containing 0.1% peptone, 0.1% yeast extract, 0.03% KCl, 0.25% MgSO_4_·7H_2_O, 0.05% CaCl_2_. Growth was evaluated by measuring the optical density at 600 nm (OD_600_) after 2 to 3 days of incubation at 150 rpm.

### Degradation ability tests for starch and protein.

Enzyme activity screenings of *Exiguobacterium* members were performed as described by Margesin et al. (79). Protease and amylase activities were tested on LB agar supplemented with skim milk (2%, wt/vol) and starch (0.4%, wt/vol). After 3 days of incubation at 25°C, the plates were scored. A reaction was determined to be positive when transparent zones were readily visible around the colonies or detected after coloration was preformed using Lugol’s iodine solution.

### Genome sequencing, assembly, and annotation.

The 105 *Exiguobacterium* strains were incubated at 25°C in LB liquid medium at 150 rpm for 2 days. Genomic DNA was extracted using a bacterial genomic DNA minikit (TaKaRa Bio) by following the manufacturer’s protocol. A paired-end library with an insert size of 350 bp was constructed for each genome and sequenced with an Illumina NovaSeq 6000 sequencer to generate 150 pair-end reads. The raw reads of each genome were processed to remove low-quality bases and adaptors by Trimmomatic v0.36 (80) and assembled with SPAdes v3.14.1 (81). The genomes of *Exiguobacterium* strains deposited in GenBank were collected and filtered based on the criterion that genomes were at least 95% complete and had <5% contamination based on CheckM v1.1.2 analysis (82). Gene predictions and annotations of all genomes were generated using Prokka v1.14.6 (83). The pairwise ANI values among all the studied genomes were computed using the FastANI v1.31 software (84).

### Phylogenetic tree construction.

Analysis of orthologous clusters was performed using FastOrtho (http://enews.patricbrc.org/fastortho/), which is a faster reimplementation of OrthoMCL (85). In brief, an all-against-all BLAST search was performed with E values of <1 × 10^−10^. Ortholog groups were created with the MCL algorithm using an inflation value of 2, and single-copy gene families were obtained using custom-made Python scripts. The protein sequences of each family were aligned using MUSCLE v3.8.1551 (86) and trimmed with trimAl v1.4.rev22 (87). All trimmed alignments were concatenated into a new alignment by a local Python script. A phylogenetic tree based on this alignment was constructed using IQ-TREE v2.0.3 with 1,000 bootstrap replicates, employing the JTT+F+I+G4 model (88). iTOL was used to visualize the phylogenetic tree (89).

### Identification of carbohydrate-active enzymes and proteases.

To identify genes encoding carbohydrate-active enzymes and proteases, all of the annotated genes were searched against the CAZy database (http://www.cazy.org) (90) and peptidase database (MEROPS) (91) using Diamond BLASTP (E value, 1E–5; ID 30; more sensitive). These carbohydrate-active enzymes were also predicted by HMMER using hidden Markov models (HMM) from dbCAN (92). Genes belonging to different carbohydrate-active enzymes or protease families were classified by in-house Python scripts according to the predictions. The potential secreted carbohydrate-active enzymes and peptidases were confirmed based on identification of extracellular transport signals, which was performed using SignalP (93).

### Identification of vital genes for environmental-stress resistance.

Each protein predicted by Prokka was annotated using BLASTP and Hmmscan against the Clusters of Orthologous Groups (COG) database and PFAM database, respectively, with E values of <1 × 10^−5^. Transporters were predicted by using Diamond BLASTP (E value, 1E–10; sensitive) to search the *Exiguobacterium* protein sequences against all Transporter Classification Database (TCDB) sequences (94).

### Data analyses.

Statistical analyses were performed using the Wilcoxon test. Correlation analyses for genome size, CDS number, and transporter number were performed using chart.Correlation from the PerformanceAnalytics package in R (https://cran.r-project.org/web/packages/PerformanceAnalytics/index.html).

### Data availability.

The genomes supporting the reported results have been deposited in GenBank under BioProject accession no. PRJNA644789 (Table S2).
