# Supplementary figures and images for: Comparative Genomics of Exiguobacterium Reveals What Makes a Cosmopolitan Bacterium
Source: mSystems. 2021 Jul 20;6(4):e00383-21. doi: 10.1128/mSystems.00383-21 (PMC8407118; doi:10.1128/mSystems.00383-21)

A

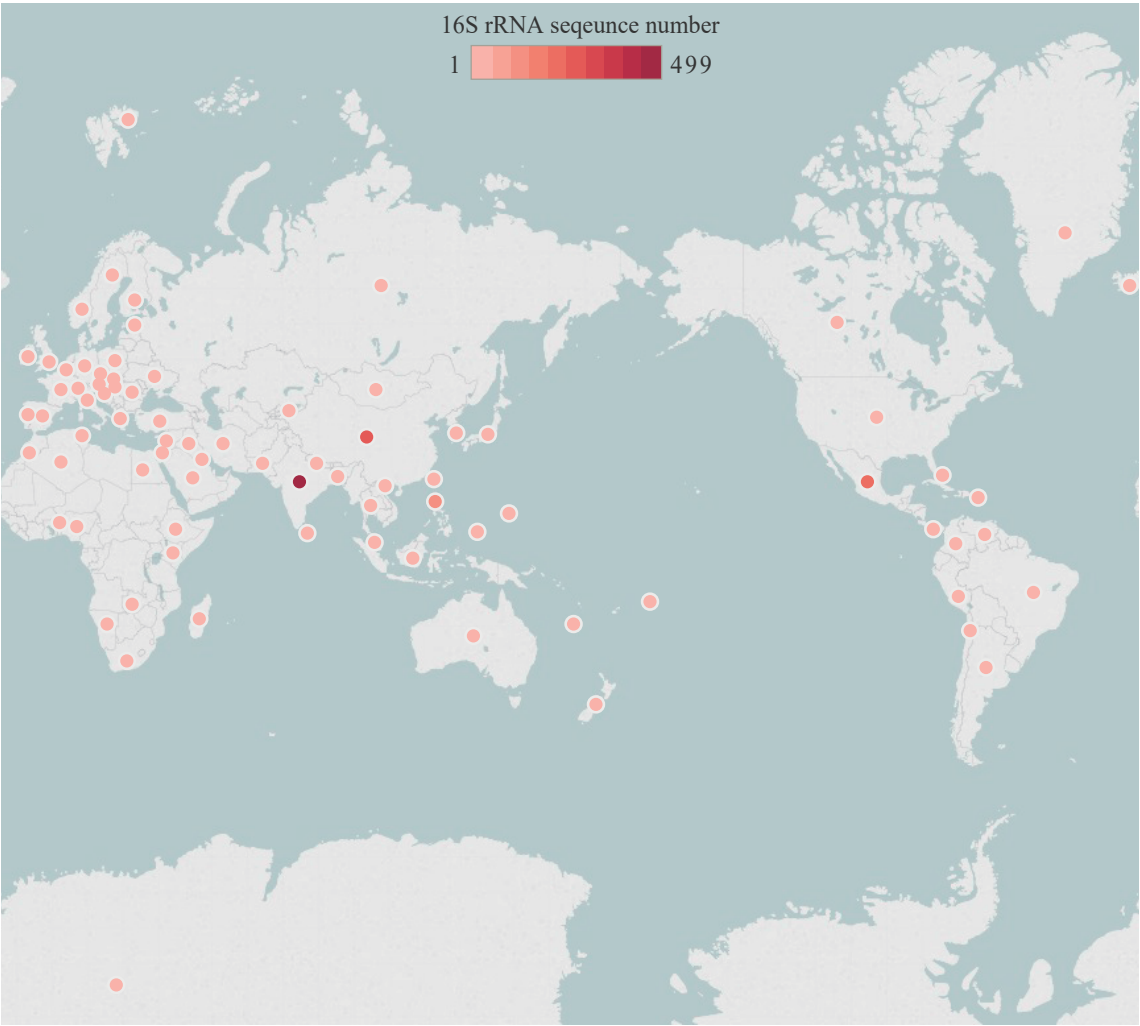

B

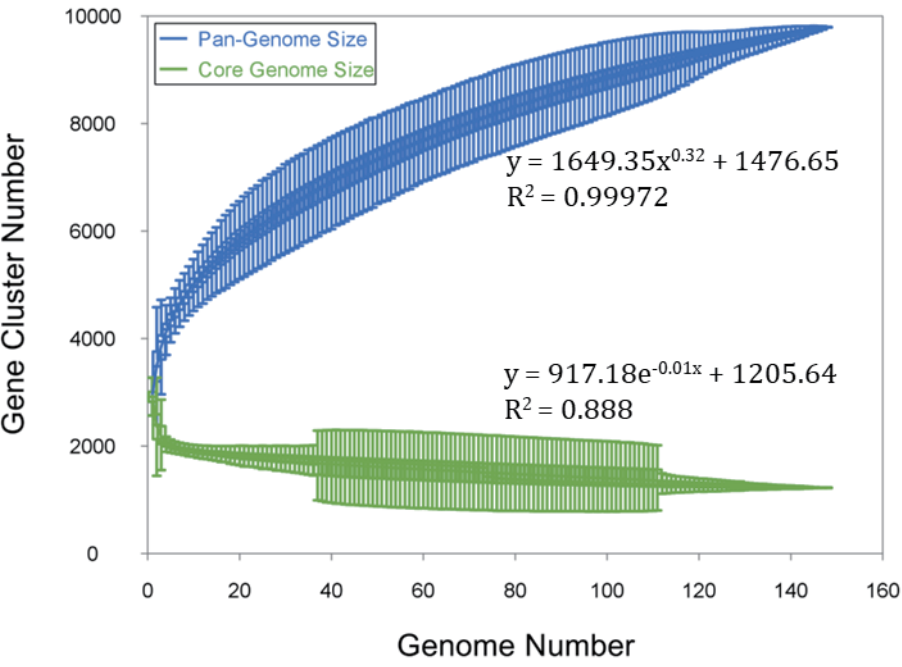

Supplement: FIG S1 [file msystems.00383-21-sf001.pdf]
